# Beyond individual markers: Prognostic value of the combined CEA/PNI score in metastatic colorectal cancer as a predictor of survival

**DOI:** 10.1371/journal.pone.0346932

**Published:** 2026-04-20

**Authors:** Edith A. Fernández Figueroa, Juan C. Falcón-Martínez, José Antonio García-Gordillo, Uriel Coquis-Navarrete, Melissa Valdés-Reyes, Haydee Williams-Sánchez, Abelardo Meneses-García, Consuelo Díaz-Romero, Marytere Herrera-Martínez, Germán Calderillo-Ruíz, Juan José Sánchez-Hernández, Erika Ruiz-García

**Affiliations:** 1 Núcleo B de Innovación en Medicina de Precisión, Instituto Nacional de Medicina Genómica, Mexico City, México; 2 Laboratorio de Medicina Traslacional, Instituto Nacional de Cancerología, Mexico City, México; 3 Instituto Nacional de Cancerología, Mexico City, México; 4 Departamento de Cirugía General, Hospital Regional “Licenciado Adolfo López Mateos”, Mexico City, México; 5 Departamento de Oncología Médica, Instituto Nacional de Cancerología, Mexico City, México; 6 Departamento de Tumores Gastrointestinales, Instituto Nacional de Cancerología, Mexico City, México; All India Institute of Medical Sciences, INDIA

## Abstract

Malnutrition and systemic inflammation significantly worsen the prognosis of oncology patients, contributing to 10–20% of cancer-related deaths. While the Carcinoembryonic Antigen (CEA) reflects tumor burden, the Prognostic Nutritional Index (PNI) captures nutritional and immune status. This study aimed to evaluate whether the combination of CEA and PNI provides superior prognostic stratification compared to either biomarker alone in patients with metastatic colorectal cancer (mCRC). This retrospective cohort study analyzed data from 512 mCRC patients at baseline and at the first response assessment (after two months of systemic treatment). Patients were stratified into four groups based on CEA (<5 or ≥5 ng/mL) and PNI (<37.52 or ≥37.52). Overall Survival (OS) and Progression-Free Survival (PFS) were analyzed using the Kaplan-Meier method and Cox regression. Our results showed that the prevalence of PNI^LOW^ was 49.8% at diagnosis and increased to 54.4% after chemotherapy (p = 0.047). PNI and Body Mass Index (BMI) status alone were not significant predictors of OS or PFS. In contrast, CEA was a powerful indicator, with CEA^HIGH^ consistently predicting worse OS and PFS. Crucially, the combined CEA/PNI score provided the strongest discrimination. Patients with the high-risk profile (CEA^HIGH^/PNI^LOW^) had a significantly increased risk of death (HR 2.41, p = 0.01) and progression (HR 2.30, p < 0.01) at baseline, compared to the lowest-risk group (CEA^LOW^/PNI^HIGH^). This elevated risk was maintained at the first assessment (HR 2.30, p < 0.01 for death and HR 6.81, p < 0.01 for progression). We conclude that PNI alone is insufficient to predict survival in mCRC patients. However, its integration with CEA creates a robust, easily obtainable biomarker that provides superior prognostic stratification by combining information on tumor burden and host systemic status.

## Introduction

Gastrointestinal tumors represent one of the leading causes of cancer-related mortality, and colorectal cancer (CRC) is the third leading cause of mortality worldwide in both men and women [1]. According to GLOBOCAN 2022, in Mexico, CRC occupies third place in incidence and first in mortality among malignant tumors in both sexes [2,3]. This notable increase in CRC in the last few years can be attributed to the change in feeding patterns, a sedentary lifestyle, and urbanization [4].

The therapeutic response to cancer depends on both intrinsic patient characteristics (such as germline genetics, the immune profile, and metabolic status) and the systemic effects induced by the tumor (including inflammation, metabolic dysregulation, and cachexia). These factors collectively exert a significant influence on the prognosis [5–7].

Up to 85% of oncology patients experience some degree of malnutrition that can affect the immune system, contributing to inferior clinical outcomes. This is attributable to multiple factors, including hyporexia, altered taste perception, nutrient malabsorption, and advanced clinical stages of the malignancy. The condition is more prevalent in gastrointestinal cancers, such as the pancreas, stomach, and colorectal cancer. Besides treatment side effects, in these malignancies, symptoms inherent to the cancer type, such as nausea, vomiting, and/or diarrhea, may also coexist [8].

Cachexia is characterized by loss of muscle and fat mass, weight loss, and systemic inflammation and is directly linked to 20% of cancer-related deaths [9]. It severely compromises prognosis, diminishing overall survival, recurrence-free survival, and quality of life; primarily by deteriorating functional status, reducing tolerance to treatment side effects, and increasing treatment toxicity, including hematological and non-hematological adverse effects. Irreversible refractory cachexia in CRC patients results in a life expectancy <3 months [9,10].

Several indices have been identified to assess nutritional status, such as Prognostic Nutritional Index (PNI), Glasgow Prognostic Score (GPS), Controlling Nutritional Status (CONUT) and some anthropometric markers like Body Mass Index (BMI). The PNI has demonstrated its prognostic utility in some solid tumors, where a low score is associated with worse clinical outcomes, shorter overall survival (OS), and poor postoperative outcomes. Furthermore, it is inexpensive and effectively evaluates immune and inflammation status in cancer patients [11,12]. Nutritional interventions improve clinical outcomes and quality of life for CRC patients. In cases requiring surgery, it has been shown that adequate preoperative and postoperative nutritional support can mitigate treatment-related complications, reduce infection rates, and expedite recovery [13]. On the other hand, carcinoembryonic antigen (CEA) is a non-specific serum biomarker frequently overexpressed in CRC, where an initial CEA level exceeding 5ng/mL at the baseline indicates a poor prognosis; a further rise suggests tumor progression [14,15]. Based on findings established in CRC stage II–III [16], the present study aimed to explore whether the combined use of CEA and PNI is superior to either biomarker alone for predicting prognosis in patients with stage IV CRC in terms of OS and progression-free survival (PFS).

## Methods

### Study design and data

This retrospective cohort study included patients treated at the Instituto Nacional de Cancerología (INCan) in México, from 01/01/2010 to 31/12/2022. Inclusion criteria were patients aged ≥18 years with stage IV CRC, adenocarcinoma, who had evaluable lesions, and regular follow-up with imaging studies and blood tests. IRB approval was obtained (No. 2022/037), and informed consent was waived. Patients were excluded if they had other primary tumors, did not receive systemic therapy, or had incomplete follow-up data. Sociodemographic, clinicopathological, and nutritional, treatment-related, laboratory, and computed tomography (CT) scan parameters, as well as survival data, were collected and analyzed. OS was defined as the time from the date of diagnosis to death from any cause or last follow-up. PFS was defined as the time from the start of systemic therapy until radiological progression, death from any cause or last follow-up. Laboratory and CT scan parameters were compared between baseline and the first systemic treatment response evaluation, performed 2 months after initiation.

### PNI and CEA

PNI was calculated using the formula: PNI= (10*serum albumin g/dL) + (0.005*total lymphocyte count per mm^3^). According to this index, patients were classified into two groups: nourished^(HIGH)^ and malnourished^(LOW)^ (where PNI^HIGH^ ≥ 37.52 and PNI^LOW^ < 37.52, respectively). The optimal cut-off was determined by analyzing their relationship with survival outcomes. CEA levels were divided into two groups: CEA^LOW^ < 5ng/mL and CEA^HIGH^ ≥ 5 ng/mL.

Patients were then classified into four groups based on their baseline and response evaluation of CEA and PNI values. Group 1: CEA^HIGH^/PNI^LOW^; Group 2: CEA^HIGH^/PNI^HIGH^; Group 3: CEA^LOW^/PNI^LOW^ and Group 4: CEA^LOW^/PNI^HIGH^.

### Statistical analysis

Descriptive and inferential statistical analyses were performed to assess data quality and explore associations. For categorical variables, absolute and relative frequencies were calculated. Continuous variables were summarized using the median and range because their distributions deviated from normality, as assessed by the Kolmogorov–Smirnov test.

Comparisons of continuous variables between independent groups were conducted using the Wilcoxon rank-sum test, while within-subject comparisons were assessed using the Wilcoxon signed-rank test. The chi-square test with continuity correction was used to compare proportions between independent dichotomous variables, and McNemar’s test was applied for paired dichotomous data.

The primary endpoints were OS and PFS. Surviving patients without evidence of progression were censored at the date of their last follow-up. Survival probabilities were estimated using the Kaplan–Meier method, and differences between cohorts were compared via the log-rank test. To evaluate the independent prognostic impact of nutritional status on both OS and PFS, multivariable Cox proportional hazards regression models were constructed. These models included candidate predictors with a univariate significance of p < 0.20 alongside clinically established confounders (age, metastatic sites, liver surgery, CT lines, CT response, CEA, ECOG). The mathematical adequacy of the Cox models was rigorously verified: the proportional hazards assumption was confirmed through Schoenfeld residual tests and visual inspection of log-log survival plots, while the linearity of continuous covariates against the log-hazard was validated using Martingale residuals. Furthermore, multicollinearity was ruled out by ensuring all Variance Inflation Factors (VIF) remained below 5. We also explored potential two-way interactions between nutritional status and other clinical variables, retaining them only if they reached statistical significance. All statistical tests were two-sided (α = 0.05) and performed using SPSS software, version 26.0 (IBM Corp., Armonk, NY).

## Results

### Clinical data

We included a total of 512 patients with stage IV CRC adenocarcinoma between 2010 and 2022. The cohort was predominantly male (56.1%) and aged over 50 years (67%). The predominant ECOG performance status observed was 1 (62%), followed by 2 (17%). Diabetes Mellitus was present in 12.7%, while arterial hypertension affected 18%. Differentiation grades 2 and 3 accounted for most cases (64.8% and 25.6%, respectively). Regarding sidedness, the left colon accounted for 83.6% of cases, within this group, the rectum was the predominant site (60.8%). Metastases were present in one site in up to 63% of cases, with the liver being the most frequent location (35.5%). Table 1 summarizes the patient demographic information.

**Table 1:** Baseline clinical and demographic characteristics of the study population (n = 512).

Variables	n = 512	%
Gender		
Women	225	43.9
Male	287	56.1
Age (years)		
Median	56 (16–85)	
Stage		
IV	512	100
Basal BMI (kg/m²)		
Underweight	20	3.9
Normal	259	50.6
Overweight	175	34.2
Obese	58	11.3
Basal PNI ^Low^		
	255	49.8
Basal CEA (ng/mL)		
<5	117	22.9
≥5	391	76.4
Presence of stoma at any time		
	212	41.4
Primary tumor sidedness		
Right	84	16.4
Left	428	83.6
Number of metastatic sites		
One site	325	63.5
Two or more sites	187	36.5
Liver resection (yes)	67	13.1
Molecular testing performed	251	100
KRAS Mutated	115	45.8
NRAS Mutated	9	3.5
BRAF Mutated	1	0.3
Systemic treatment		
≤2 lines	347	67.8
Tumor response at first assessment (CT)		
Complete	19	3.7
Partial	168	32.8
Stable	150	29.3
Progression	175	34.2

Notes: Data are presented as n (%). Median values are shown with a range. Abbreviations: BMI, body mass index; PNI low, low prognostic nutritional index; CEA, carcinoembryonic antigen; KRAS, Kirsten rat sarcoma viral oncogene homolog; NRAS, neuroblastoma RAS viral oncogene homolog; CT, computed tomography; BRAF, B-Raf proto-oncogene; CT, computed tomography; Tumor response was evaluated by computed tomography according to RECIST version 1.1 criteria: RECIST, Response Evaluation Criteria in Solid Tumors.

The median follow-up was 466 days, with a range of 50–2449 days. Regarding the first line of treatment, the regimen distribution was as follows: combination of fluoropyrimidines and oxaliplatin (85%), fluoropyrimidines monotherapy (8%), combination of fluoropyrimidines and irinotecan (6%), and triplet therapy (FOLFOXIRI) (1%). Monoclonal antibodies (antiangiogenic or antiEGFR) were used in 20% of cases. At the time of the initial assessment of systemic treatment response, 34.2% of patients had progressed. Overall, 32.2% of patients received three or more lines of chemotherapy.

### Overall survival

At diagnosis, 49.8% of patients had a PNI^LOW,^ which increased to 54.4% at the first response assessment (p = 0.047). No significant differences in OS were observed when comparing patients with PNI^LOW^ vs. PNI^HIGH^ at baseline (HR 1.16, 95% CI 0.84–1.62; p = 0.35) (S1 Table and S1 Fig). Similarly, no significant differences were found when comparing PNI^LOW^ vs. PNI^HIGH^ at the first assessment (HR 1.20, 95% CI 0.85–1.70; p = 0.28) (S2 Table and S2 Fig). Regarding CEA, significant differences in OS were observed when comparing patients with CEA^LOW^ vs. CEA^HIGH^ at baseline and at the first assessment (HR 2.27, 95% CI 1.61–4.62; p < 0.001) (HR 2.18, 95% CI 1.35–3.45; p < 0.001), respectively (S3 and S4 Tables and S3 and S4 Figs).

**Fig 1:**
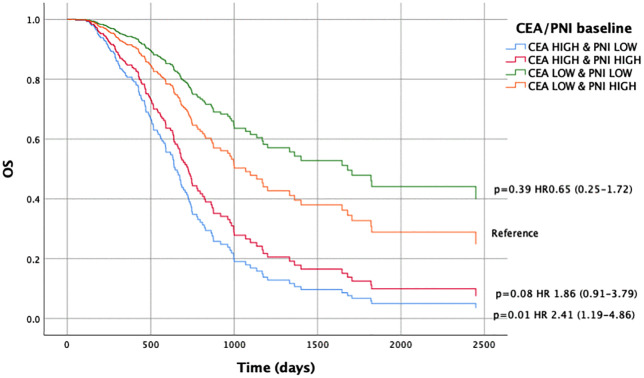
Overall survival according to the combined CEA/PNI score at baseline. Kaplan–Meier survival curves for overall survival (OS) stratified by the combined carcinoembryonic antigen (CEA) and prognostic nutritional index (PNI) score at baseline. Patients were classified into four groups: CEA^HIGH^ & PNI^LOW^, CEA^HIGH^ & PNI^HIGH^, CEA^LOW^ & PNI^LOW^, and CEA^LOW^ & PNI^HIGH^. The high-risk group (CEA^HIGH^ & PNI^LOW^) showed significantly worse OS compared to the reference group (CEA LOW & PNI HIGH), with a hazard ratio (HR) of 2.41 (95% confidence interval [CI]: 1.19–4.86; p = 0.01). Survival distributions were compared using the log-rank test.

**Fig 2:**
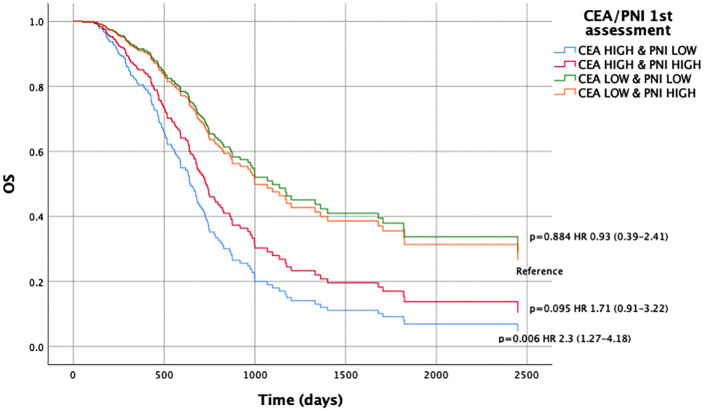
Overall survival according to the combined CEA/PNI score at first response assessment. Kaplan–Meier survival curves for overall survival (OS) stratified by the combined carcinoembryonic antigen (CEA) and prognostic nutritional index (PNI) score at the first response assessment (2 months after initiation of systemic therapy). Patients were classified into four groups: CEA^HIGH^ & PNI^LOW^, CEA^HIGH^ & PNI^HIGH^, CEA^LOW^ & PNI^LOW^, and CEA^LOW^ & PNI^HIGH^. The high-risk group (CEA^HIGH^ & PNI^LOW^) showed significantly worse OS compared to the reference group (CEA^LOW^ & PNI^HIGH^), with a hazard ratio (HR) of 2.30 (95% confidence interval [CI]: 1.27–4.18; p = 0.006). Survival distributions were compared using the log-rank test.

Concerning the CEA and PNI combination, we observed significant differences in OS when comparing patients with CEA^HIGH^/PNI^LOW^ vs. CEA^LOW^/PNI^HIGH^ at baseline (HR 2.41, 95% CI 1.19–4.86; p = 0.01) (Fig 1, S5 Table). This was also consistent at the first assessment (HR 2.30, 95% CI 1.27–4.18; p = 0.006) (Fig 2, S6 Table).

We also investigated whether being underweight vs. non-underweight (based on BMI) at baseline and at the first assessment impacted OS, finding no significant differences at either time point (HR 0.89, 95% CI 0.41–1.95; p = 0.78) (HR 1.60, 95% CI 0.93–2.98; p = 0.08) (S7 and S8 Tables and S5 and S6 Figs).

### Progression free survival

When comparing patients with PNI^LOW^ vs. PNI^HIGH^ at baseline (HR 1.26, 95% CI 0.92–1.73; p = 0.14); and at the first assessment (HR1.33, 95% CI 0.97–1.81; p = 0.07), no significant differences were observed (S9 and S10 Tables and S7 and S8 Figs). In contrast, when comparing patients with CEA^LOW^ vs. CEA^HIGH^ at baseline and at the first assessment, significant differences in PFS were observed (HR 1.60, 95% CI 1.14–2.50; p < 0.001) (HR 4.80, 95% CI 2.91–7.92; p < 0.001), respectively. (S11 and S12 Tables and S9 and S10 Figs).

**Fig 3:**
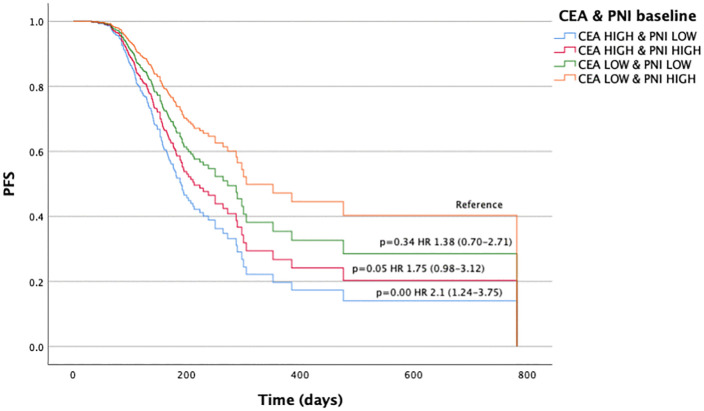
Progression-free survival according to the combined CEA/PNI score at baseline. Kaplan–Meier survival curves for progression-free survival (PFS) stratified by the combined carcinoembryonic antigen (CEA) and prognostic nutritional index (PNI) score at baseline. Patients were classified into four groups: CEA^HIGH^ & PNI^LOW^, CEA^HIGH^ & PNI^HIGH^, CEA^LOW^ & PNI^LOW^, and CEA^LOW^ & PNI^HIGH^. The high-risk group (CEA^HIGH^ & PNI^LOW^) showed significantly worse PFS compared to the reference group (CEA^LOW^ & PNI^HIGH^), with a hazard ratio (HR) of 2.16 (95% confidence interval [CI]: 1.24–3.75; p < 0.001). Survival distributions were compared using the log-rank test.

**Fig 4:**
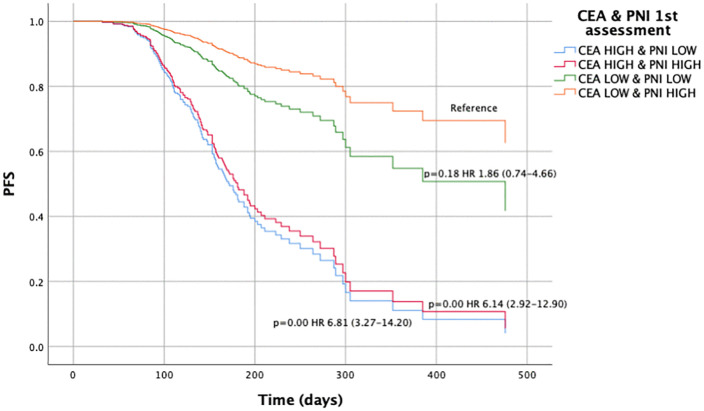
Progression-free survival according to the combined CEA/PNI score at first response assessment. Kaplan–Meier survival curves for progression-free survival (PFS) stratified by the combined carcinoembryonic antigen (CEA) and prognostic nutritional index (PNI) score at the first response assessment (2 months after initiation of systemic therapy). Patients were classified into four groups: CEA^HIGH^ & PNI^LOW^, CEA^HIGH^ & PNI^HIGH^, CEA^LOW^ & PNI^LOW^, and CEA^LOW^ & PNI^HIGH^. Both high-risk groups (CEA^HIGH^ & PNI^LOW^ and CEA^HIGH^ & PNI^HIGH^) showed significantly worse PFS compared to the reference group (CEA^LOW^ & PNI^HIGH^), with hazard ratio (HR) of 6.81 (95% confidence interval [CI]: 3.27–14.20; p < 0.001) and 6.14 (95% CI: 2.92–12.90; p < 0.001), respectively. Survival distributions were compared using the log-rank test.

Regarding PFS and the CEA/PNI combination at baseline, significant differences were observed when comparing patients with CEA^HIGH^/PNI^LOW^ (HR 2.16, 95% CI 1.24–3.75; p < 0.001) and CEA^HIGH^/PNI^HIGH^ (HR 1.75, 95% CI 0.98–3.12; p = 0.05) vs. CEA^LOW^/PNI^HIGH^ (Fig 3, S13 Table). Significant differences were also noted at the first assessment, between the same groups CEA^HIGH^/PNI^LOW^ (HR 6.81, 95% CI 3.27–14.20; p < 0.001) and CEA^HIGH^/PNI^HIGH^ (HR 6.14, 95% CI 2.92–12.90; p < 0.001) vs. CEA^LOW^/PNI^HIGH^ (Fig 4, S14 Table). (All the fully available clinical data are in S_data.sav).

Likewise, no significant differences were found in PFS between the underweight and non-underweight BMI groups at baseline (HR 1.25, 95% CI 0.61–2.56; p = 0.54) (S15 Table and S11 Fig) and at the first assessment (HR 1.57, 95% CI 0.97–2.54; p = 0.06) (S16 Table and S12 Fig).

## Discussion

The demographic landscape of CRC in Mexico reveals that the average age at diagnosis is 56 years, with approximately one-third of cases occurring in patients under 50 years of age. The majority of cases (78.1%) are diagnosed at advanced stages (III: 37.2%, and IV: 40.9%). These patients face poorer prognostic outcomes, evidenced by a 5-year OS of 58% and 33%, respectively [16]. Beyond oncologic management, it is crucial to recognize that malnutrition is associated with detrimental clinical outcomes, including compromised treatment response, diminished quality of life, and markedly elevated morbidity and mortality rates across various cancer types, including CRC. Specifically, a recent meta-analysis found that for cancer patients over 65 years, malnutrition doubled the risk of death [17]. Therefore, we designed this retrospective study to evaluate the nutritional status of our patients with metastatic CRC and investigate its impact on OS and PFS. We decided to use the BMI, PNI, and CEA because these parameters are essential for the treatment of any CRC patient and are readily available at any hospital. However, we did not incorporate other indices because they rely on metrics not routinely collected. While the PNI is calculated from albumin and total lymphocyte count, the CONUT index requires cholesterol, and the GPS score needs C-Reactive Protein (CRP) [9].

We analyzed data from 512 patients with metastatic CRC at two related time points: at baseline (diagnosis) and after the first response assessment (following two months of systemic treatment). The analysis of PNI indicated that the systemic effect of chemotherapy significantly worsened host status: the prevalence of PNI^LOW^ increased from 49.8% at diagnosis to 54.4% (a 4.6% increase) following the first two months of chemotherapy (p = 0.047). Notably, 23.6% of the total cohort underwent colostomy prior to initiating treatment. Studies indicated that colostomy is linked to a detrimental effect on nutritional status. Following surgery, patients typically lose an average of 5% of their body weight and show a marked decline in serum albumin levels within three months. These complications are often compounded by subsequent dietary changes, leading to suboptimal nutrient intake and reduced consumption of protein and fiber [18,19]. Furthermore, 85% of the patients were treated with the combination of fluoropyrimidines and oxaliplatin. This regimen’s known toxicities, which include mucositis, nausea, vomiting, and diarrhea, also have a negative impact on nutritional status [6].

The PNI, was originally designed to assess the nutritional and immunological status of patients undergoing surgery for gastrointestinal cancer; albumin reflects the host’s nutritional status, while lymphopenia is associated with impaired tumor immunity, thereby promoting tumor progression [20,21]. Although our metastatic CRC patients exhibited a negative trend in nutritional status following chemotherapy, we found that PNI alone was not sufficient to predict patient survival, despite numerous studies associating a PNI^LOW^ with poor prognosis in various cancer types [10]. Our results suggest that the lack of prognostic power for PNI may be multifaceted. First, the cut-off value for PNI remains unstandardized, lacking clear consensus across studies, and the molecular mechanisms underlying the prognostic effect of cancer still unknown [22]. Our chosen cutoff was 37.52, determined by analysis of survival outcomes; however, reported ranges in the literature vary widely from 43.65 to 48.05 [11,15,23]. Second, our findings could be influenced by uncontrolled variables inherent to this retrospective study design, including patient selection, and the heterogeneity of systemic therapies received. Finally, our results align with the literature in advanced disease; for instance, a meta-analysis in gastric cancer (GC) showed that while PNI was a reliable predictive indicator of survival and postoperative complications, a PNI^LOW^ was not significantly associated with poor OS patients with stage IV GC [24].

On the other hand, we found that in our cohort, BMI status (underweight vs. non-underweight) did not significantly predict OS or PFS at baseline. This lack of initial significance is consistent with known BMI limitations as a prognostic tool. Specifically, BMI, while measuring overall body mass, fails to differentiate between body composition (fat/muscle) or fat distribution (visceral vs. subcutaneous). This variable accuracy is further complicated by differences across sexes, age groups, and ethnicities (e.g., lower accuracy in Asian populations versus White, Black, or Mexican–American patients) [25]. The observed lack of prognostic power in our baseline data partially contrasts with the finding from Renfro et al., who analyzed over 21,000 mCRC patients and concluded that low BMI was significantly associated with an increased risk of death and progression [26]. This discrepancy may be addressed by the obesity paradox [27], a context particularly relevant since 45.5% of our cohort presented with overweight or obesity. Studies like Kocarnik et al. suggest that patients with advanced CRC who are overweight or obese have improved survival [28]. This benefit may stem from higher adiposity being advantageous for short-term metabolic demands, particularly those related to the stress of disease and the catabolic side effects of chemotherapy.

Additionally, there is a possibility that obese CRC patients have less-aggressive and less-catabolic tumors. However, the protective effect of higher BMI is likely fragile. In our study, a concerning statistical trend emerged during treatment, showing an increased risk of death and progression with subsequent weight loss. This critical observation strongly aligns with their subsequent findings [29] where they studied 2,049 CRC patients and observed that weight loss serves as a powerful indicator of poor CRC-specific survival (HR 1.25, 95% CI 1.13–1.39).

CEA has proved to be a powerful and consistent prognostic indicator. Over 75% of our patients had high basal CEA levels, and they subsequently demonstrated a significantly higher risk of death and disease progression compared to those with low levels. This finding confirms the robust prognostic value of this tumor burden marker at both baseline and the first assessment, a result consistent with earlier reports, which correlate elevated CEA levels with poor treatment response and, consequently, poor outcomes [30, 31].

Xu et al. proved that the combined use of CEA and PNI is an independent prognostic factor for OS in early stages of CRC [15]. Crucially, our study proved that the combined CEA and PNI score is a prognostic indicator for late-stage CRC, across both survival endpoints. We observed significant statistical differences in OS when comparing patients with the highest risk profile (CEA^HIGH^/PNI^LOW^) versus the lowest risk profile (CEA^LOW^/PNI^HIGH^) at baseline. This strong prognostic value was consistent at the first assessment. Regarding PFS, significant statistical differences were also observed across the combined groups at baseline, indicating that CEA^HIGH^ status negatively impacted PFS regardless of PNI. These prognostic differences were amplified at the first assessment: both the CEA^HIGH^/PNI^LOW^ and CEA^HIGH^/PNI^HIGH^ groups showed a significantly higher risk of progression. These findings show that while PNI alone is insufficient, its integration with CEA provides superior prognostic stratification by simultaneously capturing information on tumor burden and host systemic status.

The results of this research, however, must be interpreted cautiously within the context of several inherent study limitations. First, this was a retrospective, single-center study, which introduces potential selection bias and limits the generalizability of our findings to other populations. Furthermore, while the analysis controlled for several cofounders, the retrospective design implies that there may be uncontrolled variables, such as the full heterogeneity of subsequent treatment lines or specific dietary interventions, that could influence long-term survival. Second, the study utilized BMI as the sole measure for simple body composition. Although this constraint was partially addressed by employing the PNI as the primary nutritional index, the lack of more detailed body composition metrics (e.g., from CT scans) is a limitation.

## Conclusions

This study, which analyzed one of the largest cohorts of mCRC patients to date using the combined CEA/PNI score, demonstrates that this metric provides a robust prognostic stratification across OS and PFS endpoints. The high-risk profile (CEA^HIGH^/PNI^LOW^) consistently predicted the worst survival outcomes at both baseline and after the first response assessment. These findings strongly advocate for integrating nutritional interventions starting at CRC diagnosis to improve treatment outcomes and long-term quality of life.

## Supporting information

S1 Table Multivariable Cox proportional hazards model for overall survival, including baseline PNI and clinical variables. (PDF)

S2 Table Multivariable Cox proportional hazards model for overall survival, stratified by PNI at first assessment. (PDF)

S3 Table Multivariable Cox proportional hazards model for overall survival, including baseline CEA. (PDF)

S4 Table Multivariable Cox proportional hazards model for overall survival, stratified by CEA at first assessment. (PDF)

S5 Table Multivariable Cox proportional hazards model for overall survival according to combined baseline CEA and PNI groups. (PDF)

S6 Table Multivariable Cox proportional hazards model for overall survival according to combined CEA and PNI at first assessment. (PDF)

S7 Table Multivariable Cox proportional hazards model for overall survival, including baseline BMI. (PDF)

S8 Table Multivariable Cox proportional hazards model for overall survival, including BMI at first assessment. (PDF)

S9 Table Multivariable Cox proportional hazards model for progression-free survival according to baseline PNI. (PDF)

S10 Table Multivariable Cox proportional hazards model for progression-free survival according to PNI at first assessment. (PDF)

S11 Table Multivariable Cox proportional hazards model for progression-free survival according to baseline CEA. (PDF)

S12 Table Multivariable Cox proportional hazards model for progression-free survival according to CEA at first assessment. (PDF)

S13 Table Multivariable Cox proportional hazards model for progression-free survival according to combined baseline CEA and PNI groups. (PDF)

S14 Table Multivariable Cox proportional hazards model for progression-free survival according to combined CEA and PNI at first assessment. (PDF)

S15 Table Multivariable Cox proportional hazards model for progression-free survival according to baseline BMI. (PDF)

S16 Table Multivariable Cox proportional hazards model for progression-free survival, including BMI at first assessment. (PDF)

S1 Fig Kaplan–Meier curves for overall survival according to baseline PNI. Kaplan–Meier curves for overall survival (OS) by baseline prognostic nutritional index (PNI) category (low vs. high). Survival differences between groups were assessed using the log-rank test. The hazard ratio (HR), 95% confidence interval (CI), and corresponding p-value are shown in the figure.
(TIFF)

S2 Fig Kaplan–Meier curves for overall survival according to PNI at first assessment. Kaplan–Meier curves for overall survival (OS) according to prognostic nutritional index (PNI) at first assessment (low vs high). Survival differences between groups were assessed using the log-rank test. The hazard ratio (HR), 95% confidence interval (CI), and corresponding p-value are shown in the figure.
(TIFF)

S3 Fig Kaplan–Meier curves for overall survival according to baseline CEA. Kaplan–Meier curves for overall survival (OS) according to baseline CEA levels (low vs high). Survival differences between groups were assessed using the log-rank test. The hazard ratio (HR), 95% confidence interval (CI), and corresponding p-value are shown in the figure.
(TIFF)

S4 Fig Kaplan–Meier curves for overall survival according to CEA at first assessment. Kaplan–Meier curves for overall survival (OS) according to CEA at first assessment (low vs high). Survival differences between groups were assessed using the log-rank test. The hazard ratio (HR), 95% confidence interval (CI), and corresponding p-value are shown in the figure.
(TIFF)

S5 Fig Kaplan–Meier curves for overall survival according to baseline BMI. Kaplan–Meier curves for overall survival (OS) according to baseline BMI categories (underweight vs non-underweight). Survival differences between groups were assessed using the log-rank test. The hazard ratio (HR), 95% confidence interval (CI), and corresponding p-value are shown in the figure.
(TIFF)

S6 Fig Kaplan–Meier curves for overall survival according to BMI at first assessment. Kaplan–Meier curves for overall survival (OS) according to BMI at first assessment (underweight vs non-underweight). Survival differences between groups were assessed using the log-rank test. The hazard ratio (HR), 95% confidence interval (CI), and corresponding p-value are shown in the figure.
(TIFF)

S7 Fig Kaplan–Meier curves for progression-free survival according to baseline PNI. Kaplan–Meier curves for progression-free survival (PFS) according to baseline prognostic nutritional index (PNI) categories (low vs high). Survival differences between groups were assessed using the log-rank test. The hazard ratio (HR), 95% confidence interval (CI), and corresponding p-value are shown in the figure.
(TIFF)

S8 Fig Kaplan–Meier curves for progression-free survival according to PNI at first assessment. Kaplan–Meier curves for progression-free survival (PFS) according to prognostic nutritional index (PNI) at first assessment (low vs high). Survival differences between groups were assessed using the log-rank test. The hazard ratio (HR), 95% confidence interval (CI), and corresponding p-value are shown in the figure.
(TIFF)

S9 Fig Kaplan–Meier curves for progression-free survival according to baseline CEA. Kaplan–Meier curves for progression-free survival (PFS) according to baseline carcinoembryonic antigen (CEA) levels (low vs high). Survival differences between groups were assessed using the log-rank test. The hazard ratio (HR), 95% confidence interval (CI), and corresponding p-value are shown in the figure.
(TIFF)

S10 Fig Kaplan–Meier curves for progression-free survival according to CEA at first assessment. Kaplan–Meier curves for progression-free survival (PFS) according to carcinoembryonic antigen (CEA) at first assessment (low vs high). Survival differences between groups were assessed using the log-rank test. The hazard ratio (HR), 95% confidence interval (CI), and corresponding p-value are shown in the figure.
(TIFF)

S11 Fig Kaplan–Meier curves for progression-free survival according to baseline BMI. Kaplan–Meier curves for progression-free survival (PFS) according to baseline body mass index (BMI) categories (underweight vs non-underweight). Survival differences between groups were assessed using the log-rank test. The hazard ratio (HR), 95% confidence interval (CI), and corresponding p-value are shown in the figure.
(TIFF)

S12 Fig Kaplan–Meier curves for progression-free survival according to BMI at first assessment. Kaplan–Meier curves for progression-free survival (PFS) according to body mass index (BMI) at first assessment (underweight vs non-underweight). Survival differences between groups were assessed using the log-rank test. The hazard ratio (HR), 95% confidence interval (CI), and corresponding p-value are shown in the figure.
(TIFF)

S1 Data Supporting information file with all clinical data used for this manuscript. (SAV)
