# Supplementary material for: Beyond individual markers: Prognostic value of the combined CEA/PNI score in metastatic colorectal cancer as a predictor of survival
Source: PLoS One. 2026 Apr 20;21(4):e0346932. doi: 10.1371/journal.pone.0346932 (PMC13095018; doi:10.1371/journal.pone.0346932)
Supplement: S1 Table — (PDF) [file pone.0346932.s001.pdf]

S1 Table. Multivariable Cox proportional hazards model for overall survival, including baseline PNI and clinical variables.

Variable	β (B)	SE	Wald	df	p-value	HR (95% CI)
Liver surgery (yes vs no)	1.345	0.255	27.8	1	<0.001	3.838 (2.330–6.323)
CEA baseline (continuous)	1.012	0.269	14.1	1	<0.001	2.751 (1.624–4.659)
CT lines (≤2 vs ≥3)	-0.734	0.178	17.0	1	<0.001	0.480 (0.339–0.680)
CT response (responder vs non-responder)	1.067	0.182	34.2	1	<0.001	2.907 (2.033–4.157)
PNI baseline (continuous)	0.155	0.168	0.8	1	0.356	1.167 (0.840–1.622)

Abbreviations

SE, standard error; HR, hazard ratio; CI, confidence interval; CEA, carcinoembryonic antigen; CT, chemotherapy; PNI, prognostic nutritional index. P-values were calculated using the Wald test in the Cox proportional hazards model. A p-value <0.05 was considered statistically significant.
