# Supplementary material for: Beyond individual markers: Prognostic value of the combined CEA/PNI score in metastatic colorectal cancer as a predictor of survival
Source: PLoS One. 2026 Apr 20;21(4):e0346932. doi: 10.1371/journal.pone.0346932 (PMC13095018; doi:10.1371/journal.pone.0346932)
Supplement: S2 Table — (PDF) [file pone.0346932.s002.pdf]

S2 Table. Multivariable Cox proportional hazards model for overall survival, stratified by PNI at first assessment.

Variable	β (B)	SE	Wald	df	p-value	HR (95% CI)
Liver surgery (yes vs no)	1.317	0.261	25.4	1	<0.001	3.734 (2.238–6.230)
CEA baseline (continuous)	0.957	0.269	12.6	1	<0.001	2.605 (1.537–4.416)
CT lines (≤2 vs ≥3)	0.716	0.179	16.0	1	<0.001	2.046 (1.441–2.904)
CT response (responder vs non-responder)	1.076	0.181	35.3	1	<0.001	2.933 (2.057–4.181)
PNI at first assessment (continuous)	0.189	0.176	1.1	1	0.284	1.207 (0.855–1.705)

Abbreviations

SE, standard error; HR, hazard ratio; CI, confidence interval; CEA, carcinoembryonic antigen; CT, chemotherapy; PNI, prognostic nutritional index. P-values were calculated using the Wald test in the Cox proportional hazards model. A p-value <0.05 was considered statistically significant.
