# Supplementary material for: Beyond individual markers: Prognostic value of the combined CEA/PNI score in metastatic colorectal cancer as a predictor of survival
Source: PLoS One. 2026 Apr 20;21(4):e0346932. doi: 10.1371/journal.pone.0346932 (PMC13095018; doi:10.1371/journal.pone.0346932)
Supplement: S3 Table — (PDF) [file pone.0346932.s003.pdf]

S3 Table. Multivariable Cox proportional hazards model for overall survival, including baseline CEA.

Variable	β (B)	SE	Wald	df	p-value	HR (95% CI)
Liver surgery (yes vs no)	1.344	0.255	27.8	1	<0.001	3.836 (2.329–6.319)
CEA baseline (continuous)	1.007	0.268	14.1	1	<0.001	2.736 (1.618–4.627)
CT lines (≤2 vs ≥3)	-0.739	0.178	17.3	1	<0.001	0.478 (0.337–0.676)
CT response (responder vs non-responder)	1.094	0.180	36.8	1	<0.001	2.987 (2.098–4.253)

Abbreviations

SE, standard error; HR, hazard ratio; CI, confidence interval; CEA, carcinoembryonic antigen; CT, chemotherapy; PNI, prognostic nutritional index. P-values were calculated using the Wald test in the Cox proportional hazards model. A p-value <0.05 was considered statistically significant.
