# Supplementary material for: Beyond individual markers: Prognostic value of the combined CEA/PNI score in metastatic colorectal cancer as a predictor of survival
Source: PLoS One. 2026 Apr 20;21(4):e0346932. doi: 10.1371/journal.pone.0346932 (PMC13095018; doi:10.1371/journal.pone.0346932)
Supplement: S4 Table — (PDF) [file pone.0346932.s004.pdf]

S4 Table. Multivariable Cox proportional hazards model for overall survival, stratified by CEA at first assessment.

Variable	β (B)	SE	Wald	df	p-value	HR (95% CI)
Liver surgery (yes vs no)	1.101	0.250	19.4	1	<0.001	3.008 (1.844–4.907)
CT lines (≤2 vs ≥3)	-0.645	0.175	13.5	1	<0.001	0.525 (0.372–0.740)
CT response (responder vs non-responder)	0.956	0.183	27.1	1	<0.001	2.601 (1.816–3.725)
CEA at first assessment (continuous)	-0.764	0.241	10.0	1	0.002	2.180 (1.350–3.450)

Abbreviations

SE, standard error; HR, hazard ratio; CI, confidence interval; CEA, carcinoembryonic antigen; CT, chemotherapy; PNI, prognostic nutritional index. P-values were calculated using the Wald test in the Cox proportional hazards model. A p-value <0.05 was considered statistically significant.
