# Supplementary material for: Beyond individual markers: Prognostic value of the combined CEA/PNI score in metastatic colorectal cancer as a predictor of survival
Source: PLoS One. 2026 Apr 20;21(4):e0346932. doi: 10.1371/journal.pone.0346932 (PMC13095018; doi:10.1371/journal.pone.0346932)
Supplement: S5 Table — (PDF) [file pone.0346932.s005.pdf]

S5 Table. Multivariable Cox proportional hazards model for overall survival according to combined baseline CEA and PNI groups.

Variable	β (B)	SE	Wald	df	p-value	HR (95% CI)
Liver surgery (yes vs no)	1.400	0.258	29.4	1	<0.001	4.056 (2.445–6.728)
CT lines (≤2 vs ≥3)	-0.775	0.179	18.7	1	<0.001	0.461 (0.324–0.654)
CT response (responder vs non-responder)	1.042	0.183	32.3	1	<0.001	2.836 (1.980–4.060)
**CEA–PNI baseline (overall)**	—	—	15.7	3	0.001	—
├─ Group 1 vs reference	0.882	0.357	6.0	1	0.014	2.415 (1.199–4.864)
├─ Group 2 vs reference	0.621	0.364	2.9	1	0.088	1.861 (0.912–3.798)
├─ Group 3 vs reference	-0.417	0.492	0.7	1	0.396	0.659 (0.251–1.728)
ECOG (0–2 vs 3–4)	0.613	0.299	4.2	1	0.040	1.846 (1.028–3.316)

Abbreviations

SE, standard error; HR, hazard ratio; CI, confidence interval; CEA, carcinoembryonic antigen; CT, chemotherapy; PNI, prognostic nutritional index. P-values were calculated using the Wald test in the Cox proportional hazards model. A p-value <0.05 was considered statistically significant.
