# Supplementary material for: Beyond individual markers: Prognostic value of the combined CEA/PNI score in metastatic colorectal cancer as a predictor of survival
Source: PLoS One. 2026 Apr 20;21(4):e0346932. doi: 10.1371/journal.pone.0346932 (PMC13095018; doi:10.1371/journal.pone.0346932)
Supplement: S6 Table — (PDF) [file pone.0346932.s006.pdf]

S6 Table. Multivariable Cox proportional hazards model for overall survival according to combined CEA and PNI at first assessment.

Variable	β (B)	SE	Wald	df	p-value	HR (95% CI)
Liver surgery (yes vs no)	1.147	0.258	19.7	1	<0.001	3.149 (1.898–5.223)
CT lines (≤2 vs ≥3)	-0.645	0.180	12.8	1	<0.001	0.525 (0.369–0.747)
CT response (responder vs non-responder)	0.904	0.187	23.4	1	<0.001	2.470 (1.713–3.562)
ECOG (0–2 vs 3–4)	0.736	0.301	5.9	1	0.015	2.087 (1.157–3.766)
**CEA–PNI at first assessment (overall)**	—	—	11.9	3	0.008	—
├─ Group 1 vs reference	0.836	0.304	7.5	1	0.006	2.307 (1.270–4.188)
├─ Group 2 vs reference	0.538	0.322	2.7	1	0.095	1.713 (0.911–3.222)
├─ Group 3 vs reference	-0.065	0.445	0.02	1	0.884	0.937 (0.392–2.241)

Abbreviations

SE, standard error; HR, hazard ratio; CI, confidence interval; CEA, carcinoembryonic antigen; PNI, prognostic nutritional index; CT, chemotherapy; ECOG, Eastern Cooperative Oncology Group performance status. The combined CEA–PNI variable was analyzed as a categorical variable with Group 4 as the reference category. P-values were calculated using the Wald test in the Cox proportional hazards model. A p-value <0.05 was considered statistically significant.
