# Supplementary material for: Beyond individual markers: Prognostic value of the combined CEA/PNI score in metastatic colorectal cancer as a predictor of survival
Source: PLoS One. 2026 Apr 20;21(4):e0346932. doi: 10.1371/journal.pone.0346932 (PMC13095018; doi:10.1371/journal.pone.0346932)
Supplement: S7 Table — (PDF) [file pone.0346932.s007.pdf]

S7 Table. Multivariable Cox proportional hazards model for overall survival, including baseline BMI.

Variable	β (B)	SE	Wald	df	p-value	HR (95% CI)
BMI baseline (continuous)	-0.111	0.398	0.07	1	0.781	0.895 (0.411–1.951)
Liver surgery (yes vs no)	1.343	0.255	27.8	1	<0.001	3.831 (2.326–6.310)
CT lines (≤2 vs ≥3)	0.733	0.179	16.8	1	<0.001	2.082 (1.466–2.955)
CT response (responder vs non-responder)	-1.104	0.184	36.1	1	<0.001	0.331 (0.231–0.475)
CEA baseline (continuous)	1.009	0.268	14.1	1	<0.001	2.742 (1.621–4.637)

Abbreviations

SE, standard error; HR, hazard ratio; CI, confidence interval; BMI, body mass index; CEA, carcinoembryonic antigen; CT, chemotherapy. P-values were calculated using the Wald test in the Cox proportional hazards model. A p-value <0.05 was considered statistically significant.
