# Supplementary material for: Beyond individual markers: Prognostic value of the combined CEA/PNI score in metastatic colorectal cancer as a predictor of survival
Source: PLoS One. 2026 Apr 20;21(4):e0346932. doi: 10.1371/journal.pone.0346932 (PMC13095018; doi:10.1371/journal.pone.0346932)
Supplement: S8 Table — (PDF) [file pone.0346932.s008.pdf]

S8 Table. Multivariable Cox proportional hazards model for overall survival, including BMI at first assessment.

Variable	β (B)	SE	Wald	df	p-value	HR (95% CI)
Liver surgery (yes vs no)	1.380	0.257	28.9	1	<0.001	3.977 (2.405–6.576)
CT lines (≤2 vs ≥3)	0.753	0.178	17.9	1	<0.001	2.123 (1.498–3.008)
CT response (responder vs non-responder)	-1.076	0.181	35.3	1	<0.001	0.341 (0.239–0.486)
CEA baseline (continuous)	0.988	0.269	13.5	1	<0.001	2.686 (1.586–4.549)
BMI at first assessment (continuous)	0.514	0.295	3.02	1	0.082	1.672 (0.937–2.984)

Abbreviations

SE, standard error; HR, hazard ratio; CI, confidence interval; BMI, body mass index; CEA, carcinoembryonic antigen; CT, chemotherapy. P-values were calculated using the Wald test in the Cox proportional hazards model. A p-value <0.05 was considered statistically significant.
