# Supplementary material for: Beyond individual markers: Prognostic value of the combined CEA/PNI score in metastatic colorectal cancer as a predictor of survival
Source: PLoS One. 2026 Apr 20;21(4):e0346932. doi: 10.1371/journal.pone.0346932 (PMC13095018; doi:10.1371/journal.pone.0346932)
Supplement: S11 Table — (PDF) [file pone.0346932.s011.pdf]

S11 Table. Multivariable Cox proportional hazards model for progression-free survival according to baseline CEA.

Variable	β (B)	SE	Wald	df	p-value	HR (95% CI)
CT lines (≤2 vs ≥3)	-0.448	0.196	5.2	1	0.022	0.639 (0.435–0.938)
CEA baseline (continuous)	0.525	0.200	6.9	1	0.009	1.691 (1.143–2.501)

Abbreviations

SE, standard error; HR, hazard ratio; CI, confidence interval; CEA, carcinoembryonic antigen; CT, chemotherapy. P-values were calculated using the Wald test in the Cox proportional hazards model. A p-value <0.05 was considered statistically significant.
