# Supplementary material for: Beyond individual markers: Prognostic value of the combined CEA/PNI score in metastatic colorectal cancer as a predictor of survival
Source: PLoS One. 2026 Apr 20;21(4):e0346932. doi: 10.1371/journal.pone.0346932 (PMC13095018; doi:10.1371/journal.pone.0346932)
Supplement: S12 Table — (PDF) [file pone.0346932.s012.pdf]

S12 Table. Multivariable Cox proportional hazards model for progression-free survival according to CEA at first assessment.

Variable	β (B)	SE	Wald	df	p-value	HR (95% CI)
CT lines (≤2 vs ≥3)	-0.470	0.193	5.922	1	0.015	0.625 (0.428–0.913)
CEA at first assessment (continuous)	1.570	0.255	37.883	1	<0.001	4.808 (2.916–7.928)

Abbreviations
SE, standard error; HR, hazard ratio; CI, confidence interval; CEA, carcinoembryonic antigen; CT, chemotherapy. P-values were calculated using the Wald test in the Cox proportional hazards model. A p-value <0.05 was considered statistically significant.
