# Supplementary material for: Beyond individual markers: Prognostic value of the combined CEA/PNI score in metastatic colorectal cancer as a predictor of survival
Source: PLoS One. 2026 Apr 20;21(4):e0346932. doi: 10.1371/journal.pone.0346932 (PMC13095018; doi:10.1371/journal.pone.0346932)
Supplement: S13 Table — (PDF) [file pone.0346932.s013.pdf]

S13 Table. Multivariable Cox proportional hazards model for progression-free survival according to combined baseline CEA and PNI groups.

Variable	β (B)	SE	Wald	df	p-value	HR (95% CI)
**CEA–PNI baseline (overall)**	—	—	8.9	3	0.030	—
├─ Group 1 vs reference	0.772	0.281	7.5	1	0.006	2.165 (1.249–3.753)
├─ Group 2 vs reference	0.563	0.294	3.6	1	0.055	1.757 (0.988–3.123)
├─ Group 3 vs reference	0.324	0.345	0.8	1	0.347	1.383 (0.704–2.717)
CT lines (≤2 vs ≥3)	0.449	0.196	5.2	1	0.022	1.567 (1.068–2.301)

Abbreviations

SE, standard error; HR, hazard ratio; CI, confidence interval; CEA, carcinoembryonic antigen; PNI, prognostic nutritional index; CT, chemotherapy. The combined CEA–PNI variable was analyzed as a categorical variable with Group 4 as the reference category. P-values were calculated using the Wald test in the Cox proportional hazards model. A p-value <0.05 was considered statistically significant.
