# Supplementary material for: Beyond individual markers: Prognostic value of the combined CEA/PNI score in metastatic colorectal cancer as a predictor of survival
Source: PLoS One. 2026 Apr 20;21(4):e0346932. doi: 10.1371/journal.pone.0346932 (PMC13095018; doi:10.1371/journal.pone.0346932)
Supplement: S14 Table — (PDF) [file pone.0346932.s014.pdf]

S14 Table. Multivariable Cox proportional hazards model for progression-free survival according to combined CEA and PNI at first assessment.

Variable	β (B)	SE	Wald	df	p-value	HR (95% CI)
CT lines (≤2 vs ≥3)	0.466	0.193	5.8	1	0.016	1.593 (1.091–2.327)
**CEA–PNI at first assessment (overall)**	—	—	37.6	3	<0.001	—
├─ Group 1 vs reference	1.920	0.375	26.2	1	<0.001	6.819 (3.273–14.208)
├─ Group 2 vs reference	1.815	0.379	22.9	1	<0.001	6.142 (2.923–12.908)
├─ Group 3 vs reference	0.623	0.468	1.7	1	0.184	1.864 (0.744–4.669)

Abbreviations for S14 table: SE, standard error; HR, hazard ratio; CI, confidence interval; CEA, carcinoembryonic antigen; PNI, prognostic nutritional index; CT, chemotherapy. The combined CEA–PNI variable at first assessment was analyzed as a categorical variable with Group 4 as the reference category. P-values were calculated using the Wald test in the Cox proportional hazards model. A p-value <0.05 was considered statistically significant.
