# Supplementary material for: Beyond individual markers: Prognostic value of the combined CEA/PNI score in metastatic colorectal cancer as a predictor of survival
Source: PLoS One. 2026 Apr 20;21(4):e0346932. doi: 10.1371/journal.pone.0346932 (PMC13095018; doi:10.1371/journal.pone.0346932)
Supplement: S15 Table — (PDF) [file pone.0346932.s015.pdf]

**S15 Table. Multivariable Cox proportional hazards model for progression-free survival according to baseline BMI.**

| Variable | β (B) | SE | Wald | df | p-value | HR (95% CI) |
|---|---|---|---|---|---|---|
| Metastatic sites (1 vs ≥2) | 0.360 | 0.161 | 5.01 | 1 | 0.025 | 1.434 (1.046–1.966) |
| CT lines (≤2 vs ≥3) | -0.390 | 0.194 | 4.05 | 1 | 0.044 | 0.677 (0.463–0.990) |
| BMI baseline (continuous) | 0.223 | 0.366 | 0.3 | 1 | 0.542 | 1.250 (0.610–2.561) |

**Abbreviations**

SE, standard error; HR, hazard ratio; CI, confidence interval; BMI, body mass index; CT, chemotherapy. P-values were calculated using the Wald test in the Cox proportional hazards model. A p-value <0.05 was considered statistically significant.
