# Supplementary material for: Beyond individual markers: Prognostic value of the combined CEA/PNI score in metastatic colorectal cancer as a predictor of survival
Source: PLoS One. 2026 Apr 20;21(4):e0346932. doi: 10.1371/journal.pone.0346932 (PMC13095018; doi:10.1371/journal.pone.0346932)
Supplement: S16 Table — (PDF) [file pone.0346932.s016.pdf]

S16 Table. Multivariable Cox proportional hazards model for progression-free survival including BMI at first assessment

Variable	β (B)	SE	Wald	df	p-value	HR (95% CI)
Metastatic sites (1 vs ≥2)	0.359	0.161	4.9	1	0.026	1.432 (1.044–1.965)
CT lines (≤2 vs ≥3)	-0.414	0.194	4.5	1	0.033	0.661 (0.452–0.967)
BMI at first assessment (continuous)	0.453	0.245	3.4	1	0.064	1.574 (0.973–2.544)

Abbreviations

SE, standard error; HR, hazard ratio; CI, confidence interval; BMI, body mass index; CT, chemotherapy. P-values were calculated using the Wald test in the Cox proportional hazards model. A p-value <0.05 was considered statistically significant.
