# Supplementary figures and images for: Beyond individual markers: Prognostic value of the combined CEA/PNI score in metastatic colorectal cancer as a predictor of survival
Source: PLoS One. 2026 Apr 20;21(4):e0346932. doi: 10.1371/journal.pone.0346932 (PMC13095018; doi:10.1371/journal.pone.0346932)

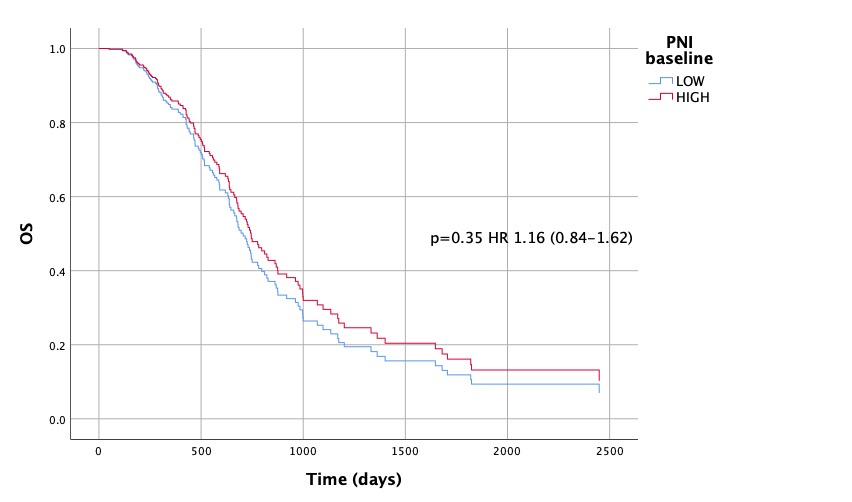

Supplement: S1 Fig — Kaplan–Meier curves for overall survival (OS) by baseline prognostic nutritional index (PNI) category (low vs. high). Survival differences between groups were assessed using the log-rank test. The hazard ratio (HR), 95% confidence interval (CI), and corresponding p-value are shown in the figure. (TIFF) [file pone.0346932.s017.TIFF]

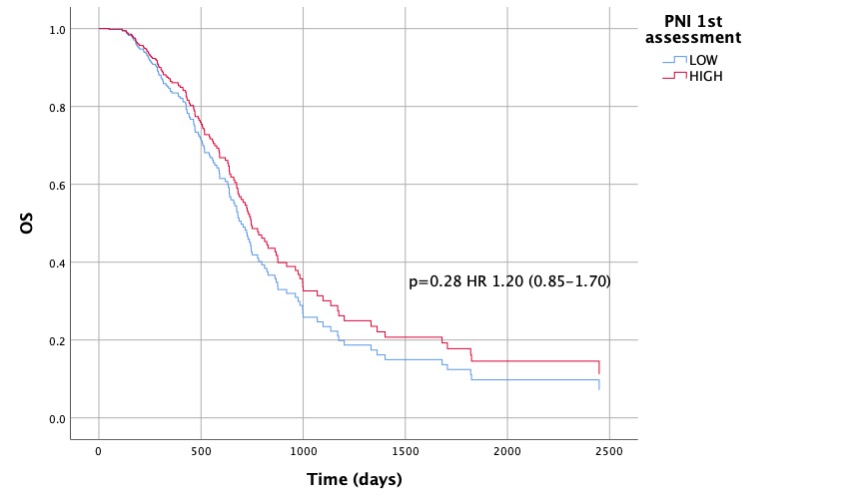

Supplement: S2 Fig — Kaplan–Meier curves for overall survival (OS) according to prognostic nutritional index (PNI) at first assessment (low vs high). Survival differences between groups were assessed using the log-rank test. The hazard ratio (HR), 95% confidence interval (CI), and corresponding p-value are shown in the figure. (TIFF) [file pone.0346932.s018.TIFF]

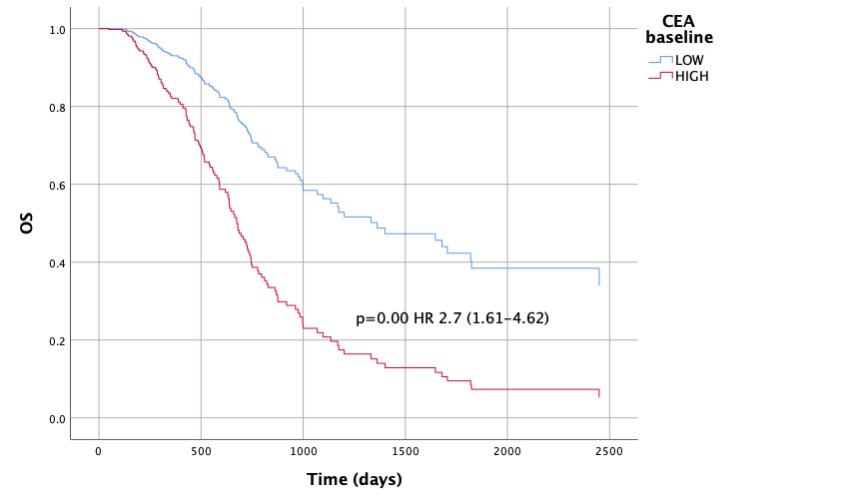

Supplement: S3 Fig — Kaplan–Meier curves for overall survival (OS) according to baseline CEA levels (low vs high). Survival differences between groups were assessed using the log-rank test. The hazard ratio (HR), 95% confidence interval (CI), and corresponding p-value are shown in the figure. (TIFF) [file pone.0346932.s019.TIFF]

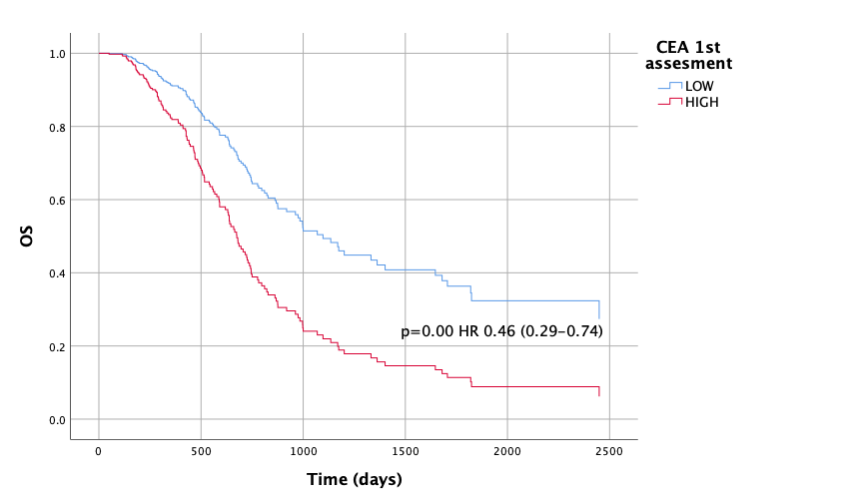

Supplement: S4 Fig — Kaplan–Meier curves for overall survival (OS) according to CEA at first assessment (low vs high). Survival differences between groups were assessed using the log-rank test. The hazard ratio (HR), 95% confidence interval (CI), and corresponding p-value are shown in the figure. (TIFF) [file pone.0346932.s020.TIFF]

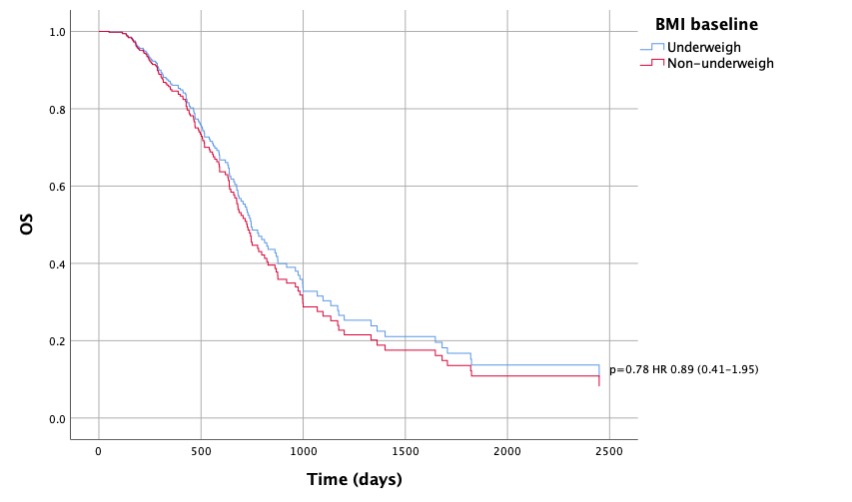

Supplement: S5 Fig — Kaplan–Meier curves for overall survival (OS) according to baseline BMI categories (underweight vs non-underweight). Survival differences between groups were assessed using the log-rank test. The hazard ratio (HR), 95% confidence interval (CI), and corresponding p-value are shown in the figure. (TIFF) [file pone.0346932.s021.TIFF]

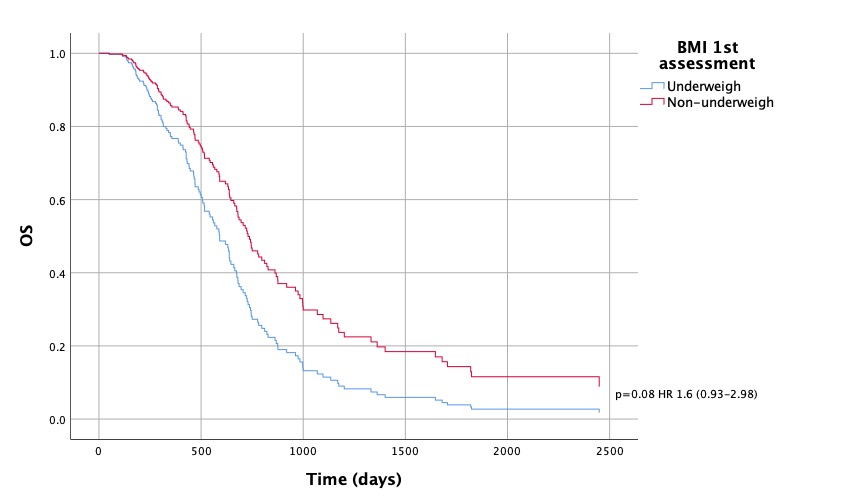

Supplement: S6 Fig — Kaplan–Meier curves for overall survival (OS) according to BMI at first assessment (underweight vs non-underweight). Survival differences between groups were assessed using the log-rank test. The hazard ratio (HR), 95% confidence interval (CI), and corresponding p-value are shown in the figure. (TIFF) [file pone.0346932.s022.TIFF]

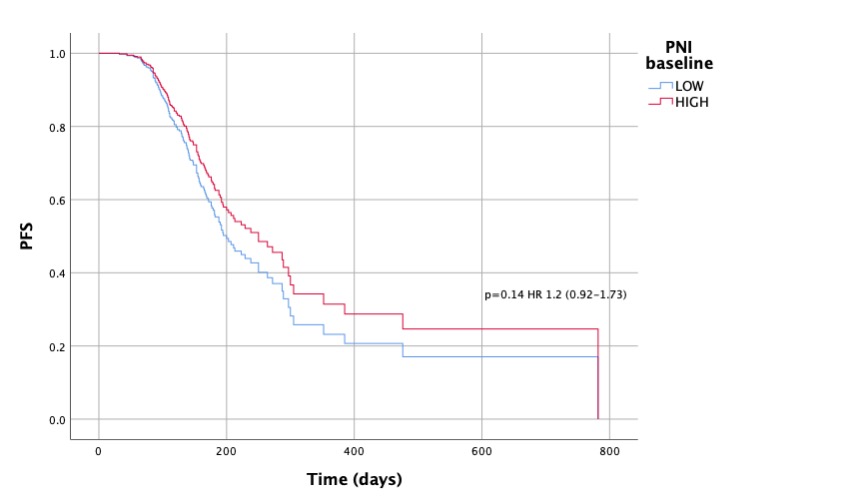

Supplement: S7 Fig — Kaplan–Meier curves for progression-free survival (PFS) according to baseline prognostic nutritional index (PNI) categories (low vs high). Survival differences between groups were assessed using the log-rank test. The hazard ratio (HR), 95% confidence interval (CI), and corresponding p-value are shown in the figure. (TIFF) [file pone.0346932.s023.TIFF]

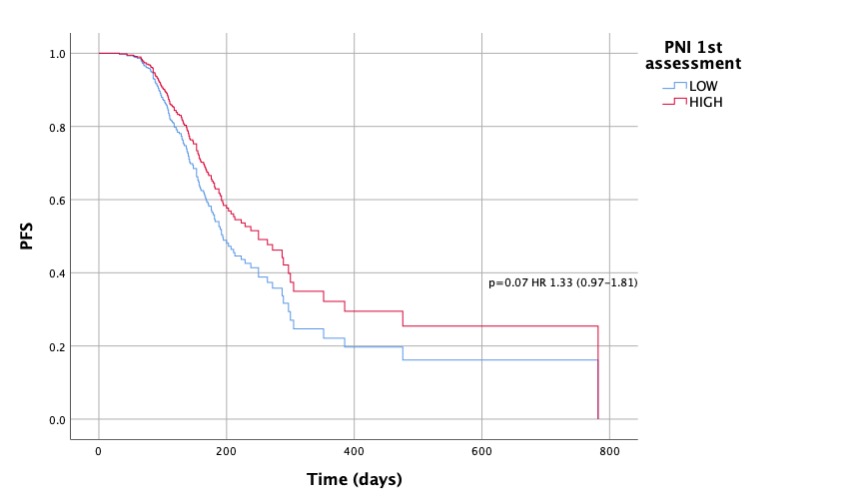

Supplement: S8 Fig — Kaplan–Meier curves for progression-free survival (PFS) according to prognostic nutritional index (PNI) at first assessment (low vs high). Survival differences between groups were assessed using the log-rank test. The hazard ratio (HR), 95% confidence interval (CI), and corresponding p-value are shown in the figure. (TIFF) [file pone.0346932.s024.TIFF]

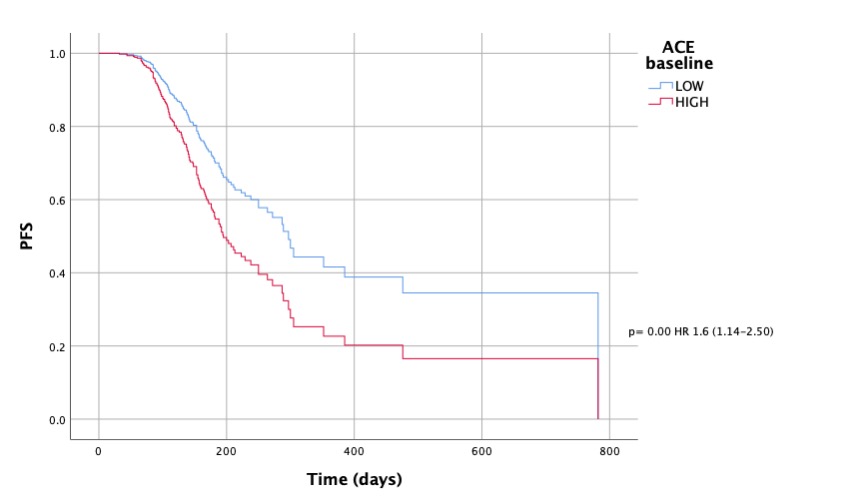

Supplement: S9 Fig — Kaplan–Meier curves for progression-free survival (PFS) according to baseline carcinoembryonic antigen (CEA) levels (low vs high). Survival differences between groups were assessed using the log-rank test. The hazard ratio (HR), 95% confidence interval (CI), and corresponding p-value are shown in the figure. (TIFF) [file pone.0346932.s025.TIFF]

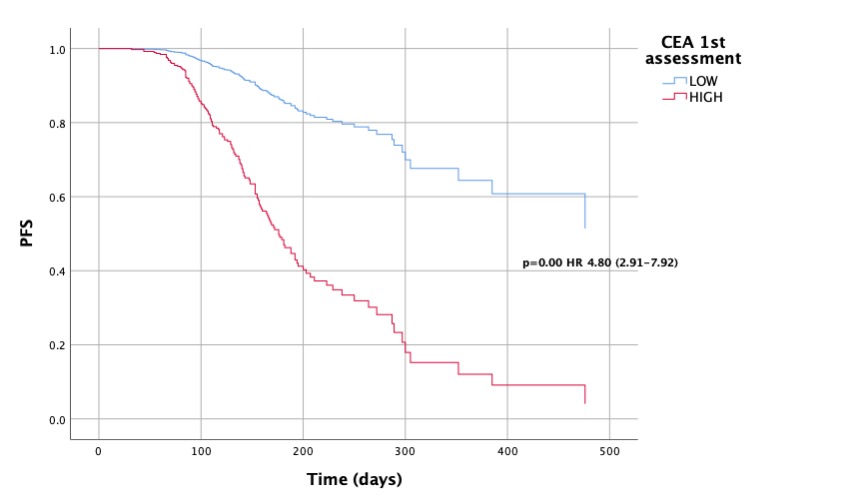

Supplement: S10 Fig — Kaplan–Meier curves for progression-free survival (PFS) according to carcinoembryonic antigen (CEA) at first assessment (low vs high). Survival differences between groups were assessed using the log-rank test. The hazard ratio (HR), 95% confidence interval (CI), and corresponding p-value are shown in the figure. (TIFF) [file pone.0346932.s026.TIFF]

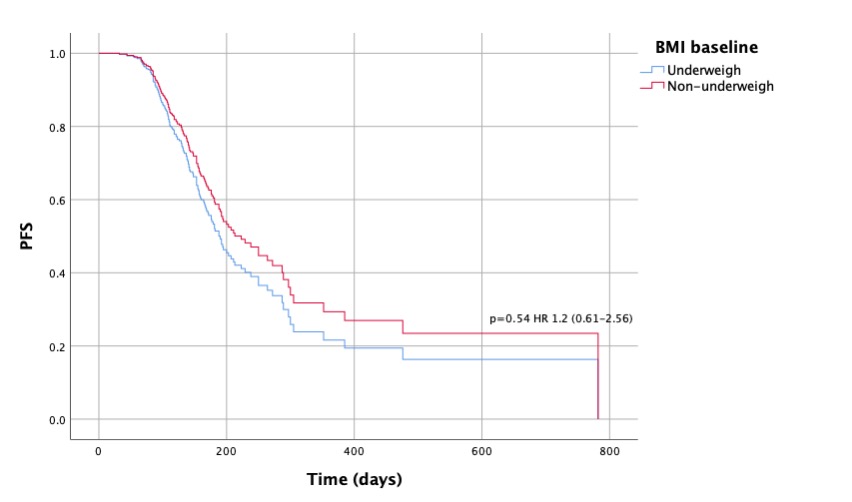

Supplement: S11 Fig — Kaplan–Meier curves for progression-free survival (PFS) according to baseline body mass index (BMI) categories (underweight vs non-underweight). Survival differences between groups were assessed using the log-rank test. The hazard ratio (HR), 95% confidence interval (CI), and corresponding p-value are shown in the figure. (TIFF) [file pone.0346932.s027.TIFF]

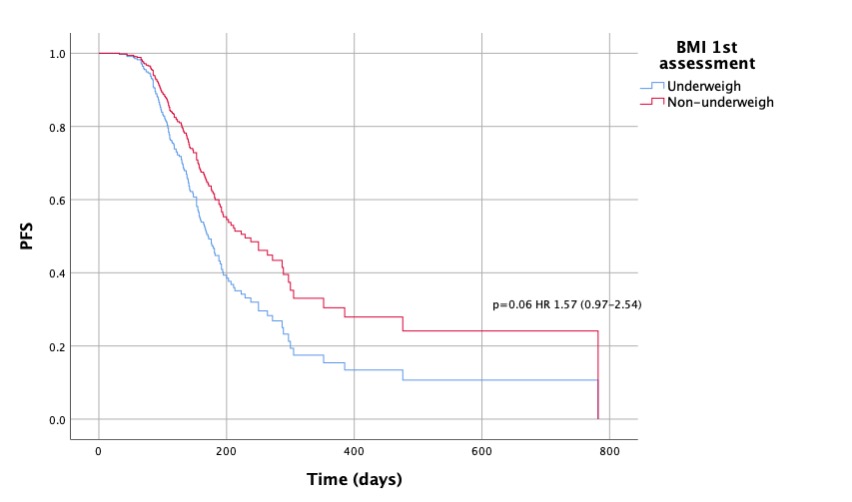

Supplement: S12 Fig — Kaplan–Meier curves for progression-free survival (PFS) according to body mass index (BMI) at first assessment (underweight vs non-underweight). Survival differences between groups were assessed using the log-rank test. The hazard ratio (HR), 95% confidence interval (CI), and corresponding p-value are shown in the figure. (TIFF) [file pone.0346932.s028.TIFF]
